# Feasibility and effectiveness of a group therapy combining physical activity, surf therapy and cognitive behavioral therapy to treat adolescents with depressive disorders: a pilot study

**DOI:** 10.3389/fpsyg.2025.1426844

**Published:** 2025-02-12

**Authors:** Bettina Hearn, Monica Biscaldi, Reinhold Rauh, Christian Fleischhaker

**Affiliations:** Department of Child and Adolescent Psychiatry, Psychotherapy, and Psychosomatics, Medical Center – University of Freiburg, Faculty of Medicine, University of Freiburg, Freiburg, Germany

**Keywords:** adolescence, depression, group therapy, physical activity, surf therapy, feasibility, effectiveness

## Abstract

**Introduction:**

The high prevalence of depression among adolescents underlines the need for further research into effective treatment options. Previous research has demonstrated the effectiveness of physical activity in reducing depressive symptoms. Recently, studies on surf therapy, as an innovative approach of physical activity, have shown promising results regarding the reduction of depressive symptoms in adults and the improvement of general mental health problems in adolescents. However, research in this area is still limited. The aim of the current study was to investigate the feasibility and effectiveness of a group therapy program that combines physical activity, including surf therapy, with cognitive behavioral therapy for treating depression among adolescents.

**Methods:**

Thirty-two outpatients (28 female, four male) aged 13–18 years with a mean age of 15.58 years (*SD* = 1.52) and a primary diagnosis of depression were included. They participated in a 3-month group therapy program in groups of eight adolescents. The dropout rate was calculated as an aspect of feasibility. To evaluate effectiveness, depressive symptoms were assessed using the “Children's Depression Rating Scale–Revised” (CDRS-R) as the primary outcome measure at pre-program, post-program, and at 3-month follow-up. In addition, questionnaires assessing depressive symptoms [“Beck Depression Inventory II” (BDI-II)], emotion regulation strategies [“Fragebogen zur Erhebung der Emotionsregulation bei Kindern und Jugendlichen” (FEEL-KJ)] and self-esteem [“Selbstwertinventar für Kinder und Jugendliche” [SEKJ)] were administered as secondary outcome measures.

**Results:**

Results showed a low dropout rate of 9.38% (*n* = 3). Depressive symptoms, assessed by the CDRS-R, were significantly reduced over time, with a large effect size. Symptom reductions were maintained at the 3-month follow-up.

**Discussion:**

Study results suggest that the group therapy program is feasible and can reduce depressive symptoms. Further research that includes control groups is needed. As a clinical implication, novel treatment forms which integrate elements of physical activity, should be considered as a treatment option for depressed adolescents

## 1 Introduction

Depressive disorders are among the most common mental illnesses in adolescence (World Health Organization, 2021). In Germany, around 6.4% of 11- to 17-year-olds show depressive symptoms (Bettge et al., 2008), with more girls than boys being affected (Mudra and Schulte-Markwort, 2020; Rao and Chen, 2009). The COVID-19 pandemic and associated restrictions have had an additional negative impact on the mental health of children and adolescents (Racine et al., 2021; Schlack et al., 2023). This is also reflected in an increase in depressive symptoms among adolescents in Germany (Naumann et al., 2021; Ravens-Sieberer et al., 2022; Schlack et al., 2023). During the sensitive developmental period of adolescence, depressive disorders can have serious negative consequences for psychological, physical and social development (Mudra and Schulte-Markwort, 2020; Naab et al., 2015; Schulte-Körne, 2023; Weissman, 1999). Depression increases the risk of comorbid psychiatric and somatic illnesses (Jonsson et al., 2011; Keenan-Miller et al., 2007; Naab et al., 2015; Small et al., 2008; Thapar et al., 2012) as well as the risk of suicidal behavior (Hawton et al., 2012; Naab et al., 2015; Thapar et al., 2012). In addition, adolescent depression increases the likelihood of further depressive episodes or chronicity in adulthood (Cook et al., 2009; Costello et al., 2002).

The S3 guideline for treating depression among adolescents in Germany is a comprehensive, evidence-based clinical guideline developed to standardize and improve the treatment of depressive disorders. It currently recommends psychotherapy [cognitive behavioral therapy (CBT) or interpersonal therapy], pharmacotherapy using fluoxetine, or the combination of CBT and fluoxetine as evidence-based treatment for depression in adolescence [Deutsche Gesellschaft für Kinder- und Jugendpsychiatrie, 2013]. This is in line with the recommendation of clinical guidelines in other countries (National Institute for Health Care Excellence, 2019). In addition, a review by Espada et al. (2023) summarizes the results of systematic reviews and meta-analyses of the last two decades on psychotherapeutic interventions for adolescent depression. CBT remains an effective approach (Klein et al., 2007; Méndez et al., 2021; Zhou et al., 2015), despite slightly discrepant results of one meta-analysis by Zhou et al. (2020), in which no consistent superiority of this approach compared to control conditions could be found.

However, outpatient psychotherapy can be associated with long waiting times, which appear to have increased further since the COVID-19 pandemic (Plötner et al., 2022). Treatment with fluoxetine may increase the risk of suicidal thoughts and actions (Bridge et al., 2007; Hammad et al., 2006).

This emphasizes the need to expand existing treatment options or to complement them with new approaches. One aspect that is discussed for optimizing care is group therapy as a form of intervention (Clayton and Burlingame, 2024; Nardi et al., 2016). The possibility of economic and standardized implementation points to the potential benefits of increased implementation of this approach. This could also help to address the growing need for mental health treatment (Clayton and Burlingame, 2024; Oei and Dingle, 2008). The effectiveness of cognitive-behavioral group therapy for treating depression in adults has already been demonstrated in a meta-analysis (Janis et al., 2021). However, the state of research for adolescents is more heterogeneous (Nardi et al., 2016). In some studies, there is evidence for the effectiveness of the group therapy approach, but there are still few randomized controlled trials for the target group of adolescents which highlights the need for further research (Nardi et al., 2016).

Another possible alternative or complementary treatment method that is increasingly being studied is physical activity (Cooney et al., 2013; Oberste et al., 2020). Meta-analyses on the effectiveness of physical activity programs for adults with depression show moderate to large effects (Cooney et al., 2013; National Institute for Health Care Excellence, 2018; Schuch et al., 2016; Stubbs et al., 2018). The achieved effects appear to be comparable to those of psychotherapy or pharmacotherapy using antidepressants (Cooney et al., 2013; Schuch et al., 2016; Stubbs et al., 2018). Low dropout rates suggest that physical activity interventions are well accepted (Stubbs et al., 2016). A recent evaluation of the intervention *STEP.De - Sports Therapy for Depression* showed that the intervention was not inferior to the standard treatment of psychotherapy in treating mild to moderate depressive disorders in adults in Germany (Gemeinsamer Bundesausschuss Innovationsausschuss, 2023b). As a result, the German Committee “Innovationsausschuss des Gemeinsamen Bundesausschusses” decided that therapeutic interventions using physical activity should be made available to adults with mild to moderate depression (Gemeinsamer Bundesausschuss Innovationsausschuss, 2023a).

Compared to the state of research in adulthood, there are significantly fewer studies on the effectiveness of physical activity on depression in adolescents. Initial meta-analyses show moderate effect sizes for physical activity interventions in reducing depressive symptoms in 12–18 year old adolescents diagnosed with depression (Bailey et al., 2018; Carter et al., 2016; Oberste et al., 2020; Wang et al., 2022). Confidence intervals range from small to large effect sizes (Bailey et al., 2018; Carter et al., 2016; Oberste et al., 2020; Wang et al., 2022). When used in clinical settings in addition to standard treatment (psychotherapy and/or pharmacotherapy), the interventions showed additional antidepressant effects (Oberste et al., 2020). In general, the reported effects are similar to those achieved in adults with depression (i.e. Stubbs et al., 2018), and to those that can be achieved by treatment approaches recommended in clinical guidelines (psychotherapy and/or pharmacotherapy) (Cipriani et al., 2016; Cuijpers et al., 2011; Klein et al., 2007; Zhou et al., 2015).

To date, only few studies have investigated the sustainability of the effects after the end of treatment. Initial results suggest that the positive effects could be maintained, i.e. 6 months after the intervention (Carter et al., 2015; Hughes et al., 2013; Oberste et al., 2018; Wunram et al., 2018). Study results also indicate good acceptance of physical activity interventions by adolescents (Bailey et al., 2018; Oberste et al., 2020; Wang et al., 2022). Dropout rates did not differ between conditions (physical activity interventions vs. control conditions) and were between 7.01% (Oberste et al., 2020), 8.33% (Wang et al., 2022) and 11% (Bailey et al., 2018). Thus, dropout rates might be even lower compared to standard treatment using psychotherapy (14.6%; 95% CI 12.0–17.4%) (Wright et al., 2021) or pharmacotherapy (23%; 95% CI 20–27%) (Rohden et al., 2017).

Regarding the characteristics of the intervention for adolescents with depression, no clear recommendations have yet been made (Bailey et al., 2018). However, comparable to the state of research among adults, aerobic activities of moderate to high intensity that are performed several times a week over a period of 8 weeks under the guidance of qualified professionals appear to lead to good results (Bailey et al., 2018; Oberste et al., 2020; Wang et al., 2022).

Overall, meta-analyses show that physical activity can reduce depressive symptoms in adolescence and could usefully supplement treatment in accordance with clinical guidelines (Bailey et al., 2018; Oberste et al., 2020; Wang et al., 2022). This is also being discussed with regard to the current revision of the German clicnial guideline for treating depression (Schulte-Körne et al., 2023). However, the validity of the studies is currently still limited due to methodological weaknesses and further studies are needed (Donath et al., 2024; Kauczor-Rieck et al., 2024; Wunram et al., 2024).

The general benefits of physical activity for mental wellbeing and physical health have already been demonstrated many times (Imboden et al., 2022; Schüler et al., 2020). With regard to the impact of physical activity on depressive symptoms, various neurobiological, psychological and psychosocial mechanisms of action are considered to exert a positive effect through a complex interaction (Bailey et al., 2018; Kauczor-Rieck et al., 2024; Wunram et al., 2024). For example, with regard to neurobiological mechanisms, exercise could transform pathological processes into regulatory mechanisms (Bailey et al., 2018; Wunram et al., 2024). One aspect that is being discussed in this regard is the influence of exercise and sport on the hypothalamic-pituitary-adrenal axis, which could reduce cortisol levels in the long term (Grandys et al., 2016; Wegner et al., 2014; Wunram et al., 2024). On a psychological level, physical activity or sport can lead to both a sense of achievement and a feeling of mastery, which in turn can strengthen the experience of self-efficacy (Bailey et al., 2018; Lubans et al., 2016; Paluska and Schwenk, 2000; Sunesson et al., 2021; Wunram et al., 2018). Exercise can also distract from negative thoughts or emotional states (Bailey et al., 2018; Paluska and Schwenk, 2000). In addition, physical activity can support an improvement in body image and body satisfaction, which in turn could contribute to a reduction in depressive symptoms (Chae et al., 2017). From a psychosocial perspective, sport that is practiced in a group can also offer the opportunity for positive social interactions (Lubans et al., 2016; Paluska and Schwenk, 2000; Wunram et al., 2024). However, the mechanisms of physical activity in relation to the reduction of depressive symptoms have not been conclusively clarified, and previous studies have shown methodological weaknesses in some cases, which emphasizes the need for further research (Kauczor-Rieck et al., 2024; Wunram et al., 2024).

Within the field of physical activity, surf therapy represents an innovative approach that utilizes the complementary benefits of integrating the element of water in nature (Barton and Pretty, 2010; Britton et al., 2020; Gascon et al., 2017). In 2019 surf therapy was defined by the International Surf Therapy Organization (ISTO) as a “method of intervention that combines surf instruction/surfing and structured individual and/or group activities to promote psychological, physical and psychosocial wellbeing” (Benninger et al., 2020). In contrast to conventional surf lessons, surf therapy provides surf instruction that is individually tailored to the participants needs with a very high level of supervision (Marshall, 2022). Surfing is complemented by other therapeutic components such as psychoeducation, group discussions, social skills training or other forms of supportive conversation (Benninger et al., 2020).

A review by Benninger et al. (2020) and a dissertation by Marshall (2022) have summarized the current state of research. Further individual studies have since been published. In adults, surf therapy is primarily being investigated in the target group of soldiers and veterans with mental disorders in the USA. Studies indicate efficacy and significant positive changes through surf therapy. These changes include reductions in depressive symptoms (Crawford, 2016; Glassman et al., 2021; Otis et al., 2020; Rogers et al., 2014; Walter et al., 2019, 2023b), post-traumatic stress disorder symptoms (Crawford, 2016; Rogers et al., 2014; Walter et al., 2019) and anxiety disorder symptoms (Glassman et al., 2021; Otis et al., 2020; Walter et al., 2023a). An increase in positive affect is also reported (Glassman et al., 2021; Otis et al., 2020; Walter et al., 2019). Regarding the significant reduction in depressive symptoms, small (Cohen's *d* = 0.42; Walter et al., 2019), moderate (Cohen's *d* = 0.77; Rogers et al., 2014) or large effect sizes (Cohen's *g* = 0.50 for proportions; Walter et al., 2023b) are reported. Regarding the decrease in symptoms of post-traumatic stress disorder, a moderate effect size is reported (Cohen's *d* = 0.61; Rogers et al., 2014). No effect sizes are available for the other publications.

For children and adolescents with mental health problems, there is evidence of significant positive changes with moderate to large effect sizes, including a reduction in mental health problems (Cohen's *d* = 0.79, Olive et al., 2023; Cohen's *d* = −0.62, Pereira et al., 2020), a reduction in behavioral problems ($\eta_{\text{p}}^{2}$ = 0.74, Gomes et al., 2020; Cohen's *d* = −0.62, Pereira et al., 2020), and improvements in wellbeing (*r* = 0.65–0.85; Marshall et al., 2021) and prosocial behavior ($\eta_{\text{p}}^{2}$ = 0.65, Gomes et al., 2020; Cohen's *d* = 0.97, Pereira et al., 2020). In addition, studies report, without stating effect sizes, a significant reduction in mental health problems (Matos et al., 2017) and significant improvements in the areas of wellbeing (Devine-Wright and Godfrey, 2020; Godfrey et al., 2015) and school (Hignett et al., 2018). Another study found positive changes in resilience, self-esteem, social connectedness and depressive symptoms through surf therapy, but did not report inferential statistics due to the small sample size and lack of power (McKenzie et al., 2021).

At present, however, the validity of the effectiveness of surf therapy is still limited. Firstly, the strength of the reported effects varies across the studies. Results with regard to various health parameters range from weak correlations or statistically insignificant improvements to significant changes (Benninger et al., 2020; Marshall, 2022). For the target group of children and adolescents, the studies of Pereira et al. (2020) and Olive et al. (2023) are the only randomized controlled studies that have been published so far. In addition, the generalizability of the results remains limited due to the heterogeneity of the target groups and of the intervention characteristics (Benninger et al., 2020; Marshall, 2022). Some of the studies also have small sample sizes and only few report follow-up data (Benninger et al., 2020; Marshall, 2022).

Besides, studies have not examined the effects of surf therapy for adolescents with formally diagnosed psychiatric disorders. However, especially for adolescents with depressive disorders, surf therapy may have therapeutic benefits. On the one hand, surf therapy has already been shown to significantly reduce depressive symptoms in adulthood (Crawford, 2016; Glassman et al., 2021; Otis et al., 2020; Rogers et al., 2014; Walter et al., 2019, 2023b). On the other hand, positive effects of physical activity on improving depressive symptoms have been demonstrated in adolescents with depression (Bailey et al., 2018; Oberste et al., 2020; Wang et al., 2022). In addition, there is no known approach to date in which surf therapy is delivered at inland locations and is therefore combined with other elements of physical activity and CBT.

To address this research gap, the aim of this study is to investigate the feasibility and effectiveness of a new group therapy program for adolescents with depression at an inland location that combines physical activity, including surf therapy, with CBT. Given the innovative nature of the group therapy, the dropout rate was chosen as an indicator to assess its feasibility, as it can demonstrate the practicality and acceptance of an approach (e.g., Stubbs et al., 2016). This helps to assess whether patients diagnosed with depression would be capable of and motivated to participate in the developed program despite the presence of depressive symptoms, such as a lack of energy.

Two hypotheses were proposed. Firstly, we hypothesized that the intervention would be feasible. This should be reflected in dropout rates comparable to previous studies on physical activity for adolescents with depression. Secondly, we hypothesized that the intervention would lead to a significant reduction in depressive symptoms over time. This should be reflected in a significant decrease in depression scores on the “Children's Depression Rating Scale–Revised” (CDRS-R, Keller et al., 2012), analyzed with a repeated measure ANOVA.

## 2 Methods

### 2.1 Participants

**Figure 1:**
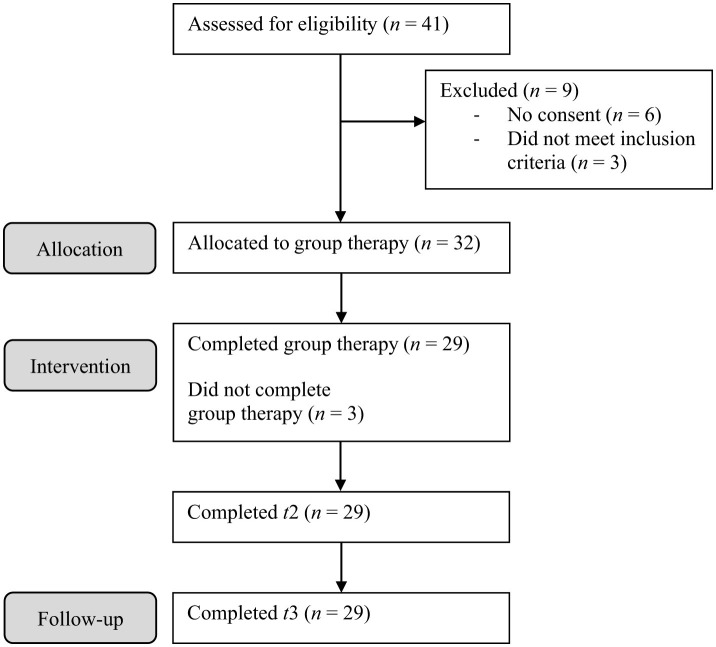
CONSORT flow chart of participants.

Thirty-two adolescents participated in the study. Forty-one adolescents were screened for participation, of which six did not agree to participate and 3 did not meet inclusion criteria (see Figure 1 for CONSORT diagram). They were recruited through the Department for Child and Adolescent Psychiatry, Psychotherapy and Psychosomatics, Medical Center—University of Freiburg from January to May of 2022 and again from January to May of 2023. The group therapy started in May of each year. To participate in the study, a primary diagnosis of mild to severe depression was required, as assessed by the diagnostic interview “Kiddie-SADS Present and Lifetime Version” (K-SADS-PL) (Delmo et al., 2001). In addition, a minimum score of 35 on the clinical interview CDRS-R (Keller et al., 2012) and a minimum score of 14 on the questionnaire “Beck Depression Inventory II” (BDI-II) (Hautzinger et al., 2009) were required. Participants had to be between 13 and 18 years old. Both male and female adolescents were included. Any concurrent pharmacotherapy or individual psychotherapy had to be kept stable throughout the group therapy. Basic swimming skills were required. Patients and their legal guardians were directly asked for this information. The participants' intellectual ability should be within the normal range, as determined by their educational background. Physical fitness for participation was tested by a general practitioner or pediatrician. Unless listed as exclusion criteria, comorbidities were possible.

As comorbid disorders we excluded acute psychosis, substance abuse, acute suicidality, bipolar disorder and severe forms of obsessive-compulsive disorder, anxiety disorder, eating disorder, autism spectrum disorders or social disorders, as assessed by the diagnostic interview K-SADS-PL (Delmo et al., 2001), which was conducted with the participants and their legal guardians separately (see Section 2.3 Procedure). Adolescents with current chronic school absenteeism or suicide attempts in the past 12 months were also excluded. Participants were not allowed to receive inpatient treatment at the same time or to participate in any other trials. If inclusion criteria were met, informed consent was obtained from the adolescents and their legal guardians.

### 2.2 Intervention

**Figure 2:**
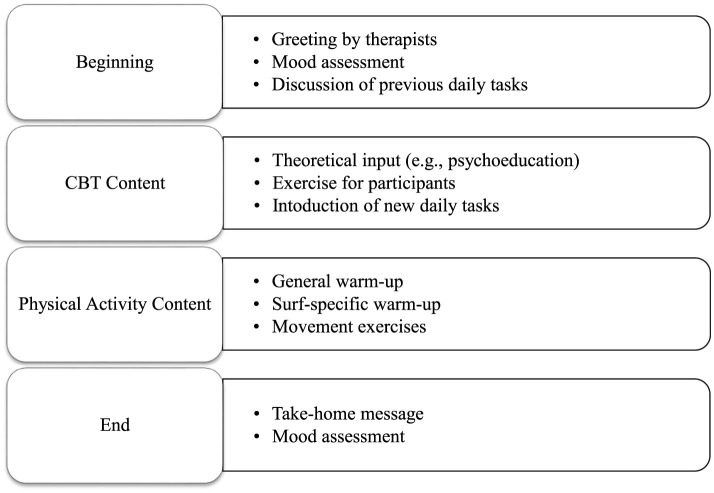
Overview of the structure of therapy sessions.

The group therapy lasted 12 weeks. Groups of 8 outpatients met once a week for 90-min sessions. Two therapists led each group, assisted by a psychology student. A therapy manual with a detailed description of all content of the group therapy as well as an accompanying workbook for the participants were developed in advance and are currently in preparation for publication. Each therapy session was a combination of physical activity and CBT. CBT is based on the concept that thoughts, emotions and actions are interconnected (Beck, 2020). By changing cognitive distortions and related behaviors this therapeutic approach aims to improve emotional regulation. During the first 11 weeks, participants were introduced to water sports including swimming, water jumping and stand-up paddling. In addition, standard elements of CBT for depression, i.e. psychoeducation, were integrated. Following each session, participants were given small assignments to transfer aspects of the group therapy content into their daily lives. Table 1 provides an overview of the group therapy content. Figure 2 summarizes the structure of the therapy sessions.

**Table 1:** Overview of the group therapy content.

**Therapy sessions**	**Therapy location**	**CBT content**	**Physical activity content**
1–3	•Gymnastics room/sports hall	•Psychoeducation on depression •Promotion of positive activities	•Introduction •Warm-up exercises •Icebreaker activities
4–9	•Indoor pool	•Resource activation •Promotion of self-esteem and emotion regulation •Improvement of crisis management	•Swimming •Diving •Jumping into water
10–11	•Lake	•Support of connection to local sports clubs	•Stand-up paddling
12–17 (intensive week)	•Coastal location with sea access	•Consolidation of previous contents •Relapse prevention	•Surf therapy

The first 11 weeks of the group therapy took place in the landlocked city of Freiburg in southern Germany. After that, the groups traveled to the North Sea (Sylt) where they stayed for 1 week and received daily surf therapy sessions in small groups of four. Each group was led by a licensed surfing instructor and a psychotherapist. In addition, two cognitive behavioral group therapy sessions took place during the week. After the intensive week of surf therapy, the group therapy ended, and the post-measurement was conducted. Three months later the follow-up assessment took place.

The project was conceptualized and realized in collaboration with the association wirmachenwelle e.V. (Managing Director: Johanna Steudtner). The Clinic for Psychiatry, Psychotherapy and Psychosomatics in Childhood and Adolescence was responsible for the organization and implementation of the therapy sessions in Freiburg. Wirmachenwelle e.V. organized the surf therapy camp at the North Sea. Together, the therapists of the clinic and the staff of wirmachenwelle e.V. realized the week of surf therapy.

Group therapy costs (e.g. travel and accommodation costs) were covered by donations (2022 Heidehof-Stiftung GmbH, 2023 wirmachenwelle e.V./Sebastian Steudtner).

### 2.3. Procedure

Data were collected at pre-program (*t*1), post-program (*t*2), and 3-month follow-up (*t*3). As pre-program assessment, the German version of the diagnostic interview “Kiddie-SADS Present and Lifetime Version” (K-SADS-PL; Delmo et al., 2001) was conducted with the participants and their legal guardians separately, to determine the current depressive episode and possible comorbidities. In addition, to evaluate the severity of the depressive symptoms in more detail, the clinical interview “Children's Depression Rating Scale–Revised” (CDRS-R) (Keller et al., 2012) was conducted, again with the participants and their legal guardians separately. Besides, questionnaires assessing depressive symptoms, emotion regulation strategies, self-esteem, and screening for general psychopathology were administered.

All participants received the same intervention. After completion, the CDRS-R and the questionnaires were administered again as post-program measurement (*t*2) and 3 months later as follow-up measurement (*t*3).

The study protocol was approved by the Ethics Committee of the University of Freiburg prior to initiation (No. 20-1137). The study was officially registered with the German Clinical Trials Register (number DRKS00024131).

### 2.4. Measures

#### 2.4.1 Primary outcome

As primary outcome measure, depressive symptom severity was assessed using the German version of the semi-structured interview “Children's Depression Rating Scale–Revised” (CDRS-R; Keller et al., 2012). Depressive symptoms are categorized into 17 areas, 14 of which are assessed by the interviewers based on the statements of the children and adolescents or their parents/ legal guardians. The assessment of the remaining three domains is based on non-verbal behavior. The 17 items are rated by the clinical interviewers on a scale of 1–5 or 1–7, where 1 describes the absence of the symptom. Total scores range from 17 to 113. A total score of at least 35 is used as a guideline for the presence of depression (Keller et al., 2012). For a clinical sample, the German version of the CDRS-R demonstrated an internal consistency of Cronbach's α = 0.90 in a validation study (Keller et al., 2011). In our study, we conducted the CDRS-R with the adolescents and their legal guardians separately and calculated a total score according to the manual (Keller et al., 2012).

#### 2.4.2 Secondary outcomes

##### 2.4.2.1 Depression

The German version of the Beck Depression Inventory II (BDI-II) (Hautzinger et al., 2009) was administered as a self-report measure to assess the severity of depressive symptoms. The questionnaire consists of 21 items using a four-point rating scale and covers all relevant symptoms of depression based on DSM-IV criteria, referring to the previous 2 weeks. Higher total scores indicate greater severity of depressive symptoms. For non-clinical samples in Germany, the questionnaire shows good psychometric parameters, with an internal consistency of Cronbach's α = 0.84 and a retest reliability of *r* ≥ 0.75 in a validation study (Kühner et al., 2007).

##### 2.4.2.2 Emotion regulation

The questionnaire “Fragebogen zur Erhebung der Emotionsregulation bei Kindern und Jugendlichen” (FEEL-KJ; Grob and Smolenski, 2009) was used to evaluate emotion regulation strategies for the emotions fear, sadness and anger. A distinction is made between adaptive strategies (problem-oriented action, distraction, mood elevation, acceptance, forgetting, reappraisal and cognitive problem solving) and maladaptive strategies (giving up, aggressive behavior, withdrawal, self-devaluation and perseveration). For the secondary scales of adaptive and maladaptive strategies, internal consistencies range from Cronbach's α = 0.82 (maladaptive strategies) to Cronbach's α = 0.93 (adaptive strategies) in a validation study. Retest reliabilities for secondary scales are at *rtt* = 0.73 (adaptive strategies) and *rtt* = 0.81 (maladaptive strategies) (Grob and Smolenski, 2009).

##### 2.4.2.3 Self-esteem

Self-esteem was measured with the questionnaire “Selbstwertinventar für Kinder und Jugendliche” (SEKJ; Schöne and Stiensmeier-Pelster, 2016). The questionnaire consists of 32 items using five-point rating scales which are combined into three scales for the three facets of self-esteem (level, stability, contingency). For school grade levels 5–6, internal consistencies are between Cronbach's α = 0.81 and α = 0.86. For school grade levels 7–10 they range from Cronbach's α = 0.87 to α = 0.90 (Schöne and Stiensmeier-Pelster, 2016). In a validation study with a 9th grade population, retest reliability (6 weeks) of the three scales range from *r* = 0.76 to *r* = 0.80 (Schöne and Stiensmeier-Pelster, 2016). It should be noted that there are no standardized values for our grade 11 participants. Standardized values for grade 10 students were applied for them as an orientation.

#### 2.4.3 Additional baseline measures

The “Child Behavior Checklist” (CBCL 6-18R; Döpfner et al., 2014), completed by parents or legal guardians, was used as a screening instrument at baseline to assess behavioral and emotional problems, somatic complaints and social skills. The “Youth Self-Report–Revised” (YSR 11-18R; Döpfner et al., 2014) was used as a corresponding self-assessment, completed by the participants. In both questionnaires the 118 items are combined into three broad-band scales (internalizing behavior problems, externalizing behavior problems, and total behavior problems) and eight narrow-band scales (anxious/depressed, withdrawn/depressed, somatic complaints, social problems, thought and repetitive problems, attention problems, rule-breaking behavior, and aggressive behavior). *T* scores can be calculated from the rawscores. The validity of the CBCL and YSR has been proven several times (Döpfner et al., 2014).

Prior to study participation, a detailed initial physical examination by the responsible general practitioner or pediatrician was carried out. This included a full blood count, ECG and EEG. Any physical contraindication to participation was to be excluded.

### 2.5 Data analysis

To determine the number of participants required for the study, a power analysis was performed using G^*^Power (Faul et al., 2007). Based on previous research, an average effect size of *d* = 0.5 was estimated. Considering a power of (1–β) = 0.80 and α = 0.05, a sample size of *N* = 27 was calculated. To account for a potential dropout rate of 15%, we calculated a sample size of *N* = 32.

Data were checked for plausibility. Descriptive statistics were computed to assess pre-program characteristics. Normality of empirical distributions were evaluated using the Shapiro–Wilk test. For the primary outcome variable (CDRS-R), a repeated measure ANOVA was conducted to examine the change of symptoms over time. Partial eta squared ($\eta_{\text{p}}^{2}$) was calculated as effect size. Effect sizes of 0.01 are considered small, 0.06 medium and effect sizes 0.14 large (Cohen, 1988). In addition to the repeated measure ANOVA, *post-hoc* pairwise comparisons were conducted using *t-*tests. For these, *d*_RepeatedMeasures_ (*d*_*RM*_) was calculated as an effect size estimate with 95% confidence intervals, which has been recommended for assessing changes within a group (Morris and DeShon, 2002). Effect sizes of 0.20 are considered small, 0.50 medium and 0.80 large (Cohen, 1988). All reported *p*-values are considered statistically significant if *p* < 0.05. Missing values were not imputed. Analyses were conducted accordingly for the BDI-II. For the other secondary outcome variables (FEEL-KJ, SEKJ) repeated measure ANOVA were calculated. Data were analyzed using SPSS software (IBM Version 28.01.0).

## 3 Results

### 3.1 Sample characteristics

Twenty-eight female and four male adolescents participated with a mean age of 15.58 years (*SD* = 1.52). As a primary diagnosis, five participants (15.63 %) met the criteria for a mild depressive episode, 14 (43.75%) for a moderate depressive episode and 13 (40.63 %) for a severe depressive episode. Fourteen participants (43.75%) had one comorbidity, eight (25%) had two and four (12.5%) had three or more comorbid diagnoses. The most common comorbidities were social phobia (*n* = 15), specific phobia (*n* = 8) and AD(H)D (*n* = 7). Table 2 presents a summary of demographic and clinical characteristics at baseline. Figure 1 displays the study's CONSORT diagram.

**Table 2:** Demographic and clinical characteristics at baseline.

	**Total sample (*N* = 32) *n* (%)**
Sex ♂	4 (12.50%)
Comorbid diagnoses	
Social Phobia (ICD-10: F40.1)	15 (46.88%)
Specific Phobia (ICD-10: F40.2)	8 (25.00%)
AD(H)D (ICD-10: F90.0)	7 (21.88%)
PTSD (ICD-10: F43.1)	3 (9.38%)
Panic disorder (ICD-10: F41.0)	3 (9.38%)
Chronic motor tic disorder (ICD-10: F95.1)	2 (6.25%)
Specific reading disorder (ICD-10: F81.0)	2 (6.25%)
Generalized Anxiety Disorder (ICD-10: F41.1)	1 (3.13%)
Obsessive-compulsive disorder (ICD-10: F42.2)	1 (3.13%)
Concurrent pharmacotherapy with selective serotonin reuptake inhibitor	6 (18.75%)
Concurrent individual psychotherapy	8 (25.00%)
	***M*** **(SD)**
Age in years	15.58 (1.52)
Pre-program measures	
CDRS-R^a^	62.91 (8.23)
BDI-II^a^	34.19 (9.55)
FEEL-KJ^b^	
Adaptive strategies	37.37 (8.43)
Maladaptive strategies	64.94 (10.43)
SEKJ^b^	
Level	34.84 (6.45)
stability	38.22 (8.06)
contingency	37.47 (9.03)
CBCL 6-18R^b^	
Internal problems	70.59 (8.77)
External problems	56.44 (11.87)
YSR 11-18R^b^	
Internal problems	76.43 (8.01)
External problems	58.47 (7.95)

CDRS-R, Children's Depression Rating Scale–Revised; BDI-II, Beck Depression Inventory II; FEEL-KJ, Fragebogen zur Erhebung der Emotionsregulation bei Kindern und Jugendlichen; SEKJ, Selbstwertinventar für Kinder und Jugendliche; CBCL 6-18R, Child Behavior Checklist; YSR 11-18R, Youth Self-Report.

^a^Rawscore.

^b^T score.

### 3.2 Feasibility

To investigate feasibility, dropouts were examined. Dropouts were defined as participants who did not complete the group therapy and therefore did not take part in the post-program or follow-up measurement. Out of the 32 participants, three adolescents had to begin inpatient treatment within the first weeks of the group therapy due to the severity of their depressive symptoms and did not complete the group therapy. Consequently, the dropout rate of our study was 9.38% (*n* = 3). Descriptively, there were no differences between the participants that dropped out and the rest of the group in age and mean values of the measurements taken at baseline. In addition, two participants did not submit all questionnaires at the follow-up assessment.

No adverse events or injuries were reported during the intervention. Furthermore, recruiting patients with depression for this type of intervention was not difficult. For all sessions the group therapy program could be carried out according to the manual.

### 3.3. Effectiveness

#### 3.3.1 Primary outcome

**Figure 3:**
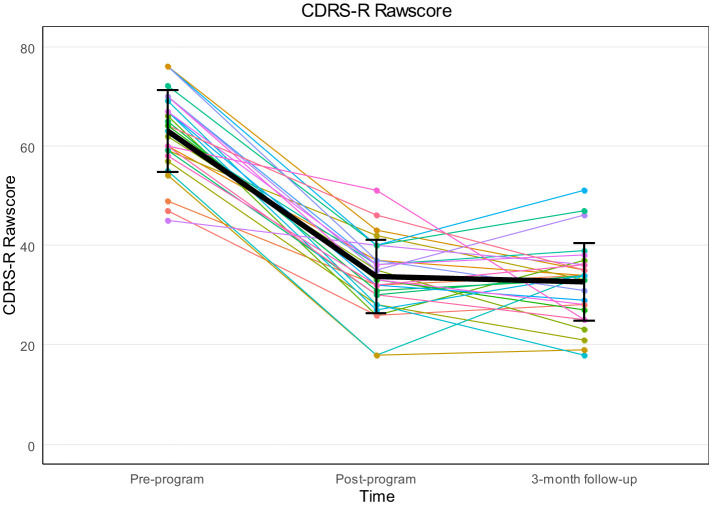
Spaghetti plot for individual courses with mean CDRS-R rawscores and standard deviation at pre-program, post-program and 3-month follow-up.

Depressive symptoms, assessed by the CDRS-R, were significantly reduced over time, with a large effect size [*F*_(2, 56)_ = 209.99, *p* < 0.001, $\eta_{\text{p}}^{2}$ = 0.882]. Sphericity assumption was met [χ^2^_(2)_ = 0.118, *p* = 0.943], no correction was necessary. Figure 3 shows the change of the CDRS-R mean scores over time. Table 3 shows the results of the repeated measure ANOVA of the CDRS-R as well as of the secondary outcome variables. *Post-hoc* tests, with Bonferroni correction, showed significant reductions between pre-program *t*1 and post-program *t*2 (*M*_1_ = 63.00, *SD*_1_ = 8.19; *M*_2_ = 33.72, *SD*_2_ = 7.4) with *p* < 0.001, *d*_*RM*_ = −3.04, and between pre-program *t*1 and 3-month follow-up *t*3 (*M*_3_ = 32.69, *SD*_3_ = 7.83) with *p* < 0.001, *d*_*RM*_ = −3.23 (see Table 4). No significant differences were found between post-program *t*2 and 3-month follow-up *t*3 (*p* = 1.00, *d*_*RM*_= −0.12).

**Table 3:** Repeated-measure ANOVA of intervention on outcome variables with $\eta_{p}^{2}$ as effect size estimate.

**Variable**	***n***	**Pre-program**		**Post-program**		**3-month follow-up**		***F***	***p***	**partial *η^2^***

		***M***	***SD***	***M***	***SD***	***M***	***SD***			
CDRS-R^a^	29	63.00	8.19	33.72	7.4	32.69	7.83	*F*_(2, 56)_ = 209.99	<0.001	0.882
BDI-II^a^	29	34.45	9.51	25.90	12.30	23.93	14.07	*F*_(2, 56)_ = 12.22	<0.001	0.304
FEEL-KJ adaptive strategies^b^	27	36.48	8.02	39.78	7.50	41.33	10.35	*F*_(2, 52)_ = 3.48	0.038	0.118
FEEL-KJ maladaptive strategies^b^	27	65.44	10.33	66.56	9.10	66.78	11.30	*F*_(1.54, 39.98)_ = 0.19	0.768	0.007
SEKJ level^b^	27	34.00	6.18	35.96	8.15	37.56	12.07	*F*_(1.51, 39.16)_ = 1.91	0.170	0.068
SEKJ stability^b^	27	38.52	8.17	41.15	8.08	42.78	10.42	*F*_(2, 52)_ = 4.15	0.021	0.137
SEKJ contingency^b^	27	37.04	8.52	36.63	8.16	38.30	10.37	*F*_(2, 52)_ = 0.62	0.544	0.023

CDRS-R, Children's Depression Rating Scale revised, BDI-II, Beck Depression Inventory II, FEEL-KJ, Fragebogen zur Erhebung der Emotionsregulation bei Kindern und Jugendlichen, SEKJ, Selbstwertinventar für Kinder und Jugendliche.

^a^Rawscore.

^b^T score.

**Table 4:** Pairwise comparisons and effect sizes for CDRS-R and BDI-II scores.

**Pairwise comparisons**		**Mean difference (I-J)**	**SE**	**Sig**.	**95% CI for difference**		**Effect size *d_*RM*_***	**95% CI for** ***d**_**RM**_*	
					**LL**	**UL**		**LL**	**UL**
CDRS-R *t*1	CDRS-R *t*2	29.28	1.71	**<**0.001	24.93	33.62	−3.04	−3.46	−1.95
CDRS-R *t*1	CDRS-R *t*3	30.31	1.71	**<**0.001	25.97	34.66	−3.23	−3.66	−2.10
CDRS-R *t*2	CDRS-R *t*3	1.03	1.62	1.00	−3.10	5.17	−0.12	−0.62	0.41
BDI-II *t*1	BDI-II *t*2	8.55	1.99	**<**0.001	3.50	13.61	−0.94	−1.38	−0.30
BDI-II *t*1	BDI-II *t*3	10.52	2.56	**<**0.001	3.99	17.05	−0.98	−1.22	−0.13
BDI-II *t*2	BDI-II *t*3	1.97	2.20	1.00	−3.64	7.57	−0.18	−0.70	0.33

Based on estimated marginal means, the mean difference is significant at the level of 0.05; Bonferroni correction applied; CDRS-R: Children's Depression Rating Scale revised, BDI-II: Beck Depression Inventory II.

t1: pre-program (baseline), t2: post-program, t3: 3-month follow-up.

At post-program, 15 (51.72%) of the 29 participants that completed the group therapy had CDRS-R scores below the cut-off of 35 for clinical relevance. At 3-month follow-up 18 of 29 (62.07%) participants reached CDRS-R scores below clinical relevance.

#### 3.3.2 Secondary outcomes

##### 3.3.2.1 Depression

**Figure 4:**
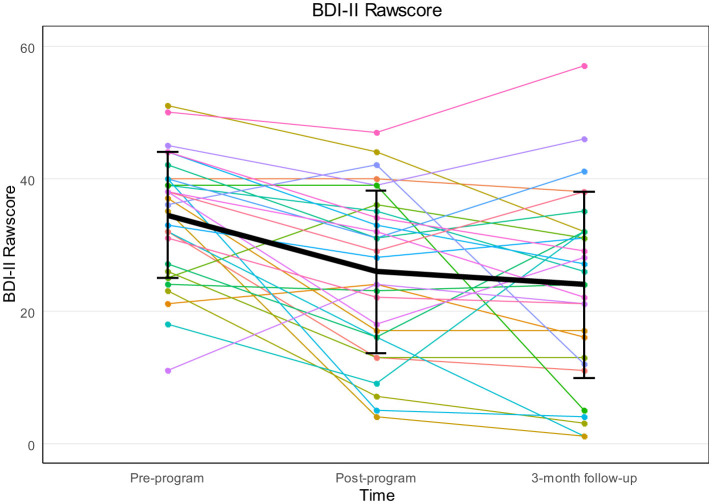
Spaghetti plot for individual courses with mean BDI-II rawscores and standard deviation at pre-program, post-program and 3-month follow-up.

Depressive symptoms, assessed by the BDI-II, were significantly reduced over time with a large effect size [*F*_(2, 56)_ = 12.22, *p* < 0.001, $\eta_{\text{p}}^{2}$ = 0.304]. Sphericity assumption was met [χ^2^_(2)_ = 2.58, *p* = 0.276]. *Post-hoc* tests, with Bonferroni correction, showed significant reductions between pre-program *t*1 and post-program *t*2 (*M*_1_ = 34.45, *SD*_1_ = 9.51; *M*_2_ = 25.90, *SD*_2_ = 12.30) with *p* < 0.001, *d*_*RM*_ = −0.94, and between pre-program *t*1 and follow-up *t*3 (*M*_3_ = 23.93, *SD*_3_ = 14.07) with *p* < 0.001, *d*_*RM*_ = −0.98. No significant differences were found between post-program *t*2 and follow-up *t*3 (*p* = 1.00, *d*_*RM*_ = −0.18). Figure 4 shows the change of the BDI-II mean scores over time.

##### 3.3.2.2 Emotion regulation

Adaptive emotion regulation strategies, assessed by the FEEL-KJ, were significantly improved over time [*F*_(2, 52)_ = 3.48, *p* = 0.038, $\eta_{\text{p}}^{2}$ = 0.118]. Sphericity assumption was met [χ^2^(2) = 5.51, *p* = 0.064]. In contrast, maladaptive emotion regulation strategies, did not change significantly over time [*F*_(1.54, 39.98)_ = 0.19, *p* = 0.768, $\eta_{\text{p}}^{2}$ = 0.007]. Sphericity assumption was not met [χ^2^_(2)_ = 8.95, *p* = 0.011] and Greenhouse-Geisser correction was performed.

##### 3.3.2.3. Self-esteem

Results of the SEKJ showed no significant changes regarding the self-esteem level over time [*F*_(1.51, 39.16)_ = 1.91, *p* = 0.170, $\eta_{\text{p}}^{2}$ = 0.068]. Sphericity assumption was not met [χ^2^_(2)_ = 9.93, *p* = 0.007], Greenhouse-Geisser correction was performed. Self-esteem contingency also did not change significantly over time [*F*_(2, 52)_ = 0.62 *p* = 0.544, $\eta_{\text{p}}^{2}$ = 0.023]. Sphericity assumption was met [χ^2^_(2)_ = 3.66, *p* = 0.161]. In contrast, self-esteem stability improved significantly over time [*F*_(2, 52)_ = 4.15, *p* = 0.021, $\eta_{\text{p}}^{2}$ = 0.137]. Sphericity assumption was met [$\chi_{\left(2\right)}^{\text{2}}$ = 2.22, *p* = 0.330].

#### 3.3.3. Exploratory analyses

Potential confounder variables were examined. Regarding the symptom reduction over time as measured by the CDRS-R, controlling for concurrent psychotherapy, concurrent pharmacotherapy (SSRI), and age did not affect the general pattern of results. They also did not affect the results of the analyses of the other measures (BDI-II, FEEL-KJ, SEKJ).

Descriptive analysis of the possible influence of concurrent pharmacotherapy on changes in the CDRS-R scores showed almost no differences at post-program between medicated participants (*n* = 6; *M*_2*med*._ = 35.50, *SD*_2*med*._ = 9.61) and unmedicated participants (*n* = 23; *M*_2*unmed*._ = 33.26, *SD*_2*unmed*._ = 6.90). However, at the follow-up measurement the participants with medication (*M*_3*med*._ = 27.17, *SD*_3*med*._ = 5.57) showed a greater symptom reduction compared to those without medication (*M*_3*unmed*._ = 34.13, *SD*_3*unmed*._ = 7.78). The interaction between the two groups and the changes in the CDRS-R scores reached significance [*F*_(2,54)_ = 3.62, *p* = 0.033, $\eta_{\text{p}}^{2}$ = 0.118]. Sphericity assumption was met [χ^2^_(2)_ = 0.89, *p* = 0.641], no correction was necessary.

## 4 Discussion

The aim of our pilot study was to investigate the feasibility and effectiveness of a group therapy program which combines physical activity, including surf therapy, with cognitive behavioral therapy for treating depression among adolescents. To the best of our knowledge, our study is the first of its kind regarding this combination of interventions for the selected target group. For example, previous studies evaluated physical activity interventions for treating depressed adolescents but did not integrate surf therapy as a form of treatment (Bailey et al., 2018; Carter et al., 2016; Oberste et al., 2020; Wang et al., 2022). Other studies focused on surf therapy but with different target groups such as depressed adults (i.e. Rogers et al., 2014; Walter et al., 2019, 2023b) or adolescents with general emotional problems (i.e. Olive et al., 2023; Pereira et al., 2020). In addition, our study is the first to integrate surf therapy in a program mostly delivered in a landlocked city and therefore implements a week of surf therapy for which the adolescents travel to the seaside.

Regarding the evaluation of feasibility of our intervention, our results show a low dropout rate of 9.38%. As hypothesized, this rate is consistent with previous reviews on physical activity for treating adolescents with depression, which reported low dropout rates between 7 and 11% (Bailey et al., 2018; Oberste et al., 2020; Wang et al., 2022). In addition, the dropout rate of our study is lower than those reported for standard treatment with psychotherapy (14.6%, Wright et al., 2021) or pharmacotherapy (23%, Rohden et al., 2017). The low dropout rate indicates that the intervention was well accepted. Despite having depression as a primary diagnosis, the adolescents were able to participate in the group therapy. Several factors may have contributed to this high level of completion. Firstly, the special intensive week of surf therapy as a new form of treatment at the end of the program might have helped motivate depressed adolescents to participate and overcome their lack of activation or possible fears regarding a group therapy. Besides, all therapists were experienced in treating depression among adolescents and created a respectful, validating, and safe environment while also accounting for the participants' individual situations or symptoms. This presumably contributed to form a good alliance between therapists and patients, which has been mentioned as beneficial regarding feasibility (Dopp et al., 2012). The relevance of creating safe spaces, specifically when delivering surf therapy, has also been shown across several studies (Marshall, 2022). In addition to the low dropout rate, we also had no difficulties recruiting patients with depression for our intervention. This has also been noted as an important aspect of feasibility (Olive et al., 2023). Moreover, the group therapy program could be carried out according to the newly developed manual.

All in all, we were able to demonstrate feasibility of our intervention. This is in line with previously published studies on the feasibility of physical activity interventions for depressed adolescents (Dopp et al., 2012; Hughes et al., 2013; Oberste et al., 2020; Wunram et al., 2018). There is less research on the feasibility of surf therapy programs in particular, but a study that focused on this aspect demonstrated feasibility as well as acceptability of a surf therapy program for children and adolescents with mental health problems (Olive et al., 2023).

Our results provide support for the effectiveness of our group therapy program for treating depression in adolescents. As hypothesized, the intervention resulted in a clinically significant reduction in depressive symptoms, as measured by the clinician-rated interview CDRS-R, considering the perspective of both the adolescents and their legal guardians. Importantly, the symptom reduction was maintained 3 months after the end of therapy. In addition to the results of the clinical interviews, the BDI-II questionnaire, as a self-report measure, also showed a large and significant decrease in symptoms. Again, the symptom reduction remained consistent at the follow-up assessment. It should be noted that the effects were sustained, despite the possibility of unfavorable external conditions during the follow-up assessment, given that it took place in November. In Germany, this month typically marks the beginning of the cold weather and can be a stressful time for adolescents due to an increase in school exams. In contrast, the post-program assessment was conducted in August at the beginning of the school summer break.

With respect to remission rates, calculated as CDRS-R scores below 35, 15 of 29 participants achieved remission after the program and 18 of 29 participants achieved remission 3 months later at the follow-up measurement. These findings contribute to the support of our intervention as a treatment approach for depression in adolescence.

Regarding secondary outcome measures, results showed a significant increase in adaptive emotion regulation strategies, but no decrease in maladaptive emotion regulation strategies. In line with the results of previous studies that postulated an association between the use of maladaptive emotion regulation strategies and depressive symptoms (Schäfer et al., 2017), the examined sample showed abnormalities in emotion regulation. Pre-measurement scores indicated infrequent use of adaptive strategies and frequent use of maladaptive strategies (see Table 2). This seemed to change only partially as a result of the group therapy. In addition, we found a significant improvement in self-esteem stability, but not in self-esteem level or self-esteem contingency. Longer periods of therapy or more specific exercises may be needed to change the complex constructs of emotion regulation and self-esteem. Exercises in individual settings may also be needed to individually address possible deficits in these areas and to initiate change. More research is necessary to evaluate and better understand the impact of the group therapy program on other psychological aspects apart from depressive symptoms.

To further interpret our findings, we compare them with previous research, taking into account possible limitations in comparability due to differences in interventions or target groups. With regard to the effects of physical activity in general, we find our results in line with previous studies which showed significant improvements in depressive symptoms among adolescents with depression (Bailey et al., 2018; Carter et al., 2016; Oberste et al., 2020; Wang et al., 2022). In addition, our results are consistent with findings on the effects of surf therapy. Previous research showed significant and large reductions in depressive symptoms among adults with depression through surf therapy (Rogers et al., 2014; Walter et al., 2019, 2023b). Regarding the target group of adolescents, studies found that surf therapy can significantly improve mental health problems (Olive et al., 2023; Pereira et al., 2020).

To date, only few studies have investigated the sustainability of the effects of physical activity interventions on depression. Consistent with our results, most studies indicate that positive changes could be sustained over time (Carter et al., 2015; Hughes et al., 2013; Oberste et al., 2018; Wunram et al., 2018). In contrast, a pilot randomized control trial on surf therapy for children and adolescents with mental health problems found that positive changes were not sustained 6 weeks after program completion (Olive et al., 2023). Further research is necessary to evaluate the sustainability of effects and to explore potential contributing factors.

In our study, most participants had comorbid diagnoses in addition to their primary diagnosis of depression. We deliberately chose to include participants with comorbidities as this reflects the typical clinical sample and therefore allows us to better draw clinical implications from the findings. Participants were also allowed to continue their individual psychotherapy or pharmacotherapy as long as they remained consistent. Exploratory data analysis indicated that the concurrent use of antidepressants may have an additional positive impact on treatment outcome. Compared to the participants not taking medication, those taking antidepressants showed a greater symptom reduction at follow-up measurement but not at post-program assessment. Walter et al. (2023b) also found that depressed adults showed greater improvements in depressive symptoms through surf or hike therapy when additionally receiving pharmacotherapy. The potential additional benefits of an antidepressant medication on the impact of the group therapy program, or the additional impact of the group therapy on pharmacotherapy, should be further investigated.

After the group therapy 17 (58.62%) participants received individual psychotherapy, 9 (31.03%) had approx. monthly psychological appointments and 3 (10.34%) did not receive further treatment. This data was collected at the follow-up measurement. It is unclear whether the weekly or monthly psychological treatment focused on depressive symptoms or on comorbidities. Further research is needed to determine in which cases the group therapy program can act as a stand-alone treatment for depression, with no need for further interventions after completion, and in which cases it is beneficial as an adjunctive treatment.

## 5 Strengths and limitations

To the best of our knowledge, this is the first study to investigate a new therapeutic approach combining physical activity including surf therapy and cognitive behavioral therapy for treating adolescents diagnosed with depression. Therefore, our study provides additional insight into this field of research with potential clinical implications. Besides, validated clinician-rated interviews as well as questionnaires were used as measurements, considering not only perspectives of the adolescents but also of their legal guardians. Data were collected at three time points, including a follow-up measurement to evaluate the sustainability of the effects which only few have done before.

The study also has some limitations which should be considered when interpreting the results. Most importantly, we did not have a control or comparison group. Hence, we cannot be certain that our results are in causal relationship with the intervention. Also, it remains unclear which elements (physical activity, surf therapy, cognitive behavioral therapy) contributed to what extent to the positive effects of the intervention. In future studies, a comparison of different group therapy aspects could be conducted. Additionally, data collection was not blinded. Mostly female patients took part which could limit the generalizability of the findings. In the future, a larger sample size with equally female and male participants could be used to investigate possible gender differences. Moreover, we focused on the assessment of change in depressive symptoms, but most participants also had comorbidities. For future studies, it would be interesting to investigate the impact of the intervention on other psychiatric symptoms. Besides, other psychological variables potentially influencing depression (i.e. anxiety or social support) were not assessed. Future research should consider these factors to better understand their role in the treatment of depressive symptoms.

## 6 Conclusion

In conclusion, our group therapy program was feasible and effective in reducing depressive symptoms among adolescents diagnosed with depression, both at post-program as well as at follow-up in comparison to the pre-program measurement. The concept of 11 weeks in a landlocked city, followed by an intensive week of surf therapy at the North Sea, was successful. The positive effect was maintained at follow-up. These results were found in a sample of patients with severe depressive symptoms and a high prevalence of comorbidities. Despite their symptoms, patients were motivated and able to participate in the intervention. The group therapy may serve as a stand-alone treatment for some adolescents with depression, while for others, it may be beneficial as an adjunctive intervention. The decision probably depends on the severity of the depressive symptoms and the presence and severity of comorbidities. As a clinical implication, novel treatment forms such as our program, which integrates elements of physical activity, should be considered as a treatment option for depressed adolescents. As a next step, a randomized controlled trial with meaningful comparison group(s) (waiting list or TAU), with a larger sample and blinded clinician rating, should be conducted to validate the results and investigate additional aspects.

## Data Availability

The datasets presented in this article are not readily available because Confidential data. Requests to access the datasets should be directed to bettina.hearn@uniklinik-freiburg.
